# Can AI write reports like a radiologist? A blinded evaluation of large language model-generated lumbar spine MRI reports

**DOI:** 10.1186/s41747-026-00682-6

**Published:** 2026-02-23

**Authors:** Moreno Zanardo, Domenico Albano, Valentina Molinari, Renato Fabrizio, Martina Conca, Luigi Asmundo, Francesco Pardo, Francesco Traina, Michele Montechiari, Salvatore Gitto, Luca Maria Sconfienza

**Affiliations:** 1 Radiology Unit, IRCCS Policlinico San Donato, San Donato Milanese, Italy; 2 Department of Radiology, ASST Grande Ospedale Metropolitano Niguarda, Milan, Italy; 3 Dipartimento di Scienze Biomediche, Chirurgiche ed Odontoiatriche, Università degli Studi di Milano, Milano, Italy; 4 Scuola di Specializzazione in Radiodiagnostica, Università degli Studi di Milano, 20122 Milan, Italy; 5 SC Ortopedia-Traumatologia e Chirurgia Protesica e dei Reimpianti d’Anca e di Ginocchio, IRCCS Istituto Ortopedico Rizzoli, Via Pupilli 1, Bologna, 40136 Italy; 6 Orthopaedics and Traumatology, University of Bologna, DIBINEM, Bologna, 40123 Italy; 7 Azienda Socio-Sanitaria Territoriale (ASST) Fatebenefratelli-Sacco, Milan, Italy; 8 IRCCS Istituto Ortopedico Galeazzi, Milan, Italy; 9 Dipartimento di Scienze Biomediche per la Salute, Università degli Studi di Milano, Milan, Italy

**Keywords:** Artificial intelligence, Diagnostic imaging, Large language models, Magnetic resonance imaging, Spine

## Abstract

**Background:**

To compare the quality and clinical usefulness of large language model (LLM)-generated lumbar spine magnetic resonance imaging (MRI) reports with radiologist-written ones and assess whether medical professionals can distinguish between them.

**Materials and methods:**

This retrospective observational single-center study was approved by the local ethics committee. A total of 125 lumbar spine MRI reports (104 human-written, 21 LLM-generated using ChatGPT-4o) were anonymized, randomized, and blindly evaluated by five medical professionals (one board-certified radiologist, two radiology residents, one general practitioner, one orthopedic surgeon), all with basic familiarity with LLM. Each report was scored on a five-point Likert scale for clinical relevance, clarity, completeness, diagnostic accuracy, and intelligibility, whereas general practitioner and orthopedic surgeon evaluated intelligibility only. Evaluators also classified each report as AI-generated or human-written. Accuracy was defined as the proportion of correctly classified reports in distinguishing LLM-generated from radiologist-written texts. Mann-Whitney *U* or Student's *t-*tests were used.

**Results:**

Radiologists' reports consistently received higher median scores across all domains (*p* < 0.001). No differences were found in the description of the imaging technique (*p* > 0.175). No clinically false statements were identified in the LLM-generated reports. Identification accuracy varied widely among evaluators: Board-certified radiologist achieved 88.0% accuracy (sensitivity 66.7%, specificity 92.3%), Resident 1 65.6% (14.3%, 76.0%), Resident 2 94.4% (66.7%, 100%), orthopedic surgeon 78.4% (90.5%, 76.0%) and general practitioner 65.6% (81.0%, 62.5%).

**Conclusion:**

Radiologist-written lumbar spine MRI reports outperform LLM-generated reports in quality and structure. However, some AI-generated reports were indistinguishable from human ones, particularly for non-specialized readers. LLMs may support radiologists in structured reporting and improve workflow efficiency, while maintaining diagnostic reliability.

**Relevance statement:**

Large language models can draft lumbar spine MRI reports, but currently lack the quality and consistency of radiologist reports. With radiologist supervision, large language models may improve reporting efficiency while preserving diagnostic reliability and supporting clinical decision-making.

**Key Points:**

- LLM-generated reports are clinically coherent and stylistically comparable to those written by expert radiologists.
- Radiologist-written reports scored significantly higher for clinical relevance, findings, and structure.
- LLM-generated reports were sometimes misclassified as human-written by clinicians.

**Graphical Abstract:**

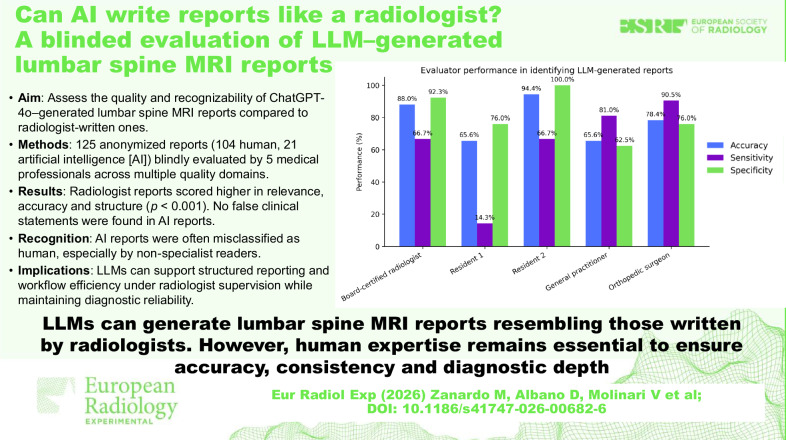

## Introduction

The rapid advancement of generative artificial intelligence (AI) has led to the widespread use of large language models (LLMs), commonly known as “chatbots” and based on the Transformer architecture introduced by Vaswani et al (2017) [1]. Tools such as Chat Generative Pre-Trained Transformer (ChatGPT) by OpenAI and Gemini by Google are prominent examples of these technologies. These AI systems are designed to understand context, interpret information, and generate coherent, human-like text across various domains [2–5]. In the medical field, growing interest surrounds their application in diagnostic support and radiology workflows [6, 7], particularly in the evaluation and reporting of imaging studies such as magnetic resonance imaging (MRI) scans [8–13].

MRI is widely used for evaluating spinal conditions, especially in the lumbosacral region, where disorders such as degenerative disc disease, spinal stenosis, herniated discs, and trauma are commonly observed [14]. These conditions are major contributors to disability and pain, affecting millions of individuals worldwide [15].

For lumbar spine MRI, a well-organized report typically includes the clinical indication, a description of the imaging technique used and a detailed account of imaging findings [16, 17]. It is also recommended that these reports follow established guidelines provided by radiological societies and musculoskeletal imaging standards [18]. Because of this standardized structure, lumbar spine MRI reporting is well-suited to integration with LLMs, which can be trained to generate structured content from specific clinical inputs [19].

LLMs can assist radiologists by helping draft MRI reports [20, 21]. This approach may reduce the time spent on documentation, increase report consistency, and help lower the risk of human error, especially in settings with high imaging volumes [22, 23].

The goal of this study is to compare the quality and clinical usefulness of LLM-generated radiology reports with those written by experienced radiologists. It also aims to determine whether radiologists can distinguish between AI-generated and human-written reports. In addition, the study evaluates whether general practitioners and orthopedic surgeons, the main receivers of lumbar spine MRI reports, can detect differences in content or style between reports created by radiologists and those generated by LLMs. This evaluation will help determine the practicality of using LLMs in routine clinical radiology and assess whether AI-generated structured reports can support efficiency while maintaining reporting quality.

## Materials and methods

### Study design and ethical approval

This retrospective observational study was conducted in accordance with the Declaration of Helsinki (as revised in 2013). The study was approved by the Institutional Review Board (Comitato Etico Territoriale Lombardia 1, RETRORAD, CE: 61/INT/2017, 12^th^ March 2025). Informed consent was waived due to the retrospective nature of the study.

### Study population

The study included adult patients of any sex who underwent lumbar spine MRI for any clinical indication at the Radiology Unit of IRCCS Ospedale Galeazzi-Sant’Ambrogio, Milan, between January 2024 and June 2024. Only exams acquired within the institution using the standardized lumbar spine protocol were considered. MRI studies were excluded if the acquisition protocol was incomplete, if image artifacts hindered diagnostic interpretation, or if the imaging was performed at an external institution.

### MRI image acquisition

All lumbar spine MRI examinations were performed using a 1.5-T MRI scanner (Signa, GE Healthcare). The institutional protocol included sagittal and axial T2-weighted turbo spin-echo sequences, sagittal short-tau inversion recovery, and sagittal T1-weighted turbo spin-echo sequences, ensuring comprehensive evaluation of lumbar spine structures.

### Data extraction and report generation

Radiology reports were retrospectively retrieved from the internal hospital database. Reports included in the analysis were authored by five different board-certified radiologists with varying years of experience in musculoskeletal imaging (from 3 years to 15 years) and selected on the basis of completeness and clinical relevance. Both the clinical indications, when available, and the narrative content of each report were extracted and compiled into a structured database for further assessment. All textual data were completely anonymized prior to their introduction into ChatGPT, with removal of any patient-, operator-, or institution-specific identifiers in compliance with applicable data protection standards. In addition to the original radiologist reports, a set of AI-generated reports was created using ChatGPT-4o (OpenAI). These were based on predefined clinical scenarios constructed by the research team (L.A., V.M., and M.Z.) to mirror common real-world lumbar spine imaging indications. The generation of these reports followed standardized prompting procedures, with input consisting of relevant clinical details, imaging techniques and findings. The clinical information used to generate the reports consisted of the referral reason for MRI as recorded in the institutional radiology information system. Both datasets comprised routine lumbar spine MRI studies covering the typical range of musculoskeletal conditions, including low back pain, lumbar disc herniation, degenerative disc disease, spinal canal or foraminal stenosis, postoperative follow-up, suspected spondylolisthesis, and spinal tumors (primary or secondary). This composition ensured a realistic representation of lumbar spine pathologies encountered in daily practice.

Each prompt explicitly instructed the model to adhere to the same structured reporting format used for radiologist-written reports. Radiologists’ original reports also followed this standardized institutional template. Since LLMs statistically emulate input data and are not recursive, some variability in phrasing and level of detail is inevitable even when the same structure is imposed. This approach ensured that raters could not rely on formal or structural elements to distinguish between human and AI-generated reports, focusing instead on the perceived clarity and quality of content. The model did not have access to the MRI images or any additional patient data. Each AI-generated report was manually checked by two radiologists and one researcher with experience in AI before anonymization to identify hallucinations or implausible statements: none were observed. Full prompt scripts are provided in the Supplementary Material, where complete examples of the text used to generate each report are included. All reports, both human- and AI-generated, were fully anonymized, randomized and evaluated independently under blinded conditions to ensure objective assessment and avoid any identification of report origin.

### Report evaluation

All raters were fully blinded to the origin of each report and were not informed about the number or proportion of AI-generated *versus* radiologist-written reports included in the dataset. The entire set of 125 reports was presented in random order through a standardized anonymized template to prevent any recognition bias. Raters did not have access to the corresponding MRI images or to any predefined list of imaging findings. They evaluated only the anonymized textual reports. All reports were written and evaluated in Italian. All raters were native Italian speakers, ensuring that language did not influence report assessment. Each report was independently reviewed by five medical professionals: one board-certified radiologist with 2 years of clinical experience (L.A.), two senior radiology residents (M.C., R.F.), one general practitioner with 11 years of clinical experience (M.M.) and one orthopedic surgeon with 10 years of experience (F.P.). All raters had only a basic, non-specialized familiarity with LLMs, reflecting general exposure to publicly available tools, but no formal training or professional experience with AI-assisted reporting systems. All five reviewers had never trained or worked at the institution where the reports were originally written.

The board-certified radiologist and the radiology residents evaluated each report using six standardized criteria: relevance of clinical indications, clarity of imaging technique description, completeness of imaging findings, accuracy of impressions or conclusions, intelligibility for radiologists and adherence to structured reporting guidelines. The items “completeness of imaging findings” and “accuracy of impressions” were assessed based only on the internal coherence, level of detail and comprehensiveness of the description with respect to the examination type, without image-based verification. The general practitioner and orthopedic surgeon assessed each report based only on its intelligibility for non-radiologist physicians. This design reflects the typical approach of structured report quality assessment studies focusing on linguistic and content quality rather than diagnostic accuracy. All items were rated on a five-point Likert scale, where 1 represented poor quality or minimal adherence and 5 indicated excellent quality or full adherence. Each evaluator was also asked to classify each report as either AI-generated or radiologist-written.

### Statistical analysis

Descriptive statistics were computed for each evaluation item by aggregating the scores across all raters. Depending on data distribution, either the mean and standard deviation or the median and interquartile range was reported. Normality was assessed using visual inspection and standard tests. Depending on the distribution, Student's *t* (for normally distributed data) or Mann-Whitney *U* tests (for non-normally distributed data) were applied to compare evaluation scores between radiologist-written and LLM-generated reports, as well as among different evaluator groups. Reports were categorized based on their actual source, radiologist or AI-generated and evaluator classifications were used to calculate sensitivity, specificity, accuracy and error rates. A true positive was defined as an AI-generated report correctly identified as such, while a true negative referred to a radiologist-written report accurately recognized. Misclassifications were categorized as either false positives or false negatives. All tests were two-tailed, and a *p*-value < 0.05 was considered statistically significant.

## Results

A total of 104 consecutive lumbar spine MRI reports, authored by five different board-certified radiologists (around 20 cases per radiologist) with 3 to 15 years of experience in musculoskeletal imaging at IRCCS Ospedale Galeazzi-Sant’Ambrogio, Milan, Italy, were retrospectively extracted from the internal database. Clinical indications, when available, and the full body text were extracted from each radiological report and transferred into an Excel database for evaluation.

In addition to the original reports, 21 new lumbar spine MRI reports were generated using ChatGPT-4o, based on predefined sets of clinical information that were formulated by the research team to simulate realistic reporting conditions.

The 21 LLM-generated reports were produced to represent an additional “virtual sixth radiologist” with a comparable number of cases. This design ensured a balanced number of reports per author/source and helped mitigate heterogeneity among human raters. No clinically incorrect or contradictory statements were identified in any of the AI-generated reports. Altogether, a total of 125 reports (104, 83.2% original radiologist reports and 21, 16.8% AI-generated reports) were included. All reports were anonymized to remove any identifying information regarding the radiologist or patient.

### Evaluator performance summary

Table 1 presents sensitivity, specificity, accuracy and error rates for each reader in distinguishing between LLM-generated and radiologist-written lumbar spine MRI reports.

**Table 1:** Performance metrics of each evaluator in identifying LLM-generated *versus* radiologist-written lumbar spine MRI reports

Reader	TP	FP	FN	TN	Sensitivity (%)	Specificity (%)	Accuracy (%)	Error rate (%)
Board-certified radiologist	14	8	7	96	66.7	92.3	88.0	15/125 (12.0)
Resident 1	3	25	18	79	14.3	76.0	65.6	43/125 (34.4)
Resident 2	14	0	7	104	66.7	100	94.4	7/125 (5.6)
General practitioner	17	39	4	65	81.0	62.5	65.6	43/125 (34.4)
Orthopedic surgeon	19	25	2	79	90.5	76.0	78.4	27/125 (21.6)

The table reports true positives (TP), false positives (FP), false negatives (FN), and true negatives (TN), along with sensitivity, specificity, overall accuracy, and error rate

The board-certified radiologist correctly identified most report origins, with a sensitivity of 66.7% and a specificity of 92.3%, corresponding to an overall accuracy of 88.0% and an error rate of 15/125 (12%).

Resident 1 demonstrated the lowest sensitivity (14.3%) and moderate specificity (76.0%), with an overall accuracy of 65.6%. The high error rate (34.4%) suggests difficulty in correctly identifying LLM-generated reports, leading to frequent misclassification as human-written (high false-negative rate).

Resident 2 showed the most accurate discrimination, with no false positives, resulting in a specificity of 100% and a sensitivity of 66.7%, leading to the highest accuracy of 94.4% and a very low error rate (5.6%). This indicates a high ability to correctly identify both AI and human-written reports.

The general practitioner achieved high sensitivity (81%) but at the cost of lower specificity (62.5%), often misidentifying radiologist reports as AI-generated (39 false positives). The overall accuracy was 65.6%, with an error rate of 34.4%, similar to Resident 1.

The orthopedic surgeon had the highest sensitivity of all (90.5%) and a balanced specificity (76.0%), with an accuracy of 78.4% and an error rate of 21.6%, indicating relatively strong performance in distinguishing between the two types of reports.

The board-certified radiologist achieved high overall accuracy (88.0%), confirming consistent discrimination between human- and AI-generated reports. Radiology residents showed contrasting results, with Resident 2 significantly outperforming Resident 1. Among non-radiologist evaluators, the orthopedic surgeon demonstrated better overall performance than the general practitioner.

### Reader-specific assessment of report quality

Board-certified radiologists and both radiology residents consistently rated radiologist-written reports significantly higher than LLM-generated ones across all major criteria, including clinical indications, imaging findings, impressions, intelligibility, and overall guideline adherence.

Board-certified radiologist (Table 2) assigned overall higher scores to radiologist-written reports, particularly emphasizing their superior structure and diagnostic coherence, with median values of 4 [4] for relevant clinical information and 4 [3, 4] for impressions. In contrast, LLM-generated reports received notably lower scores, with medians of 2 [2–4] for clinical indications and 2 [1–3] for imaging findings. No significant differences were found in the evaluation of examination technique and procedures (*p* = 0.294), indicating that this aspect was perceived as comparably handled by both human and AI authors.

**Table 2:** Evaluation of report quality by the board-certified radiologist

Board-certified radiologist	LLMs-generated score median [range]	Radiologist-written score median [range]	Significance
Relevant clinical indications/information	2 [2–4]	4 [4]	*p* < 0.001
Examination technique and procedures	1 [1]	1 [1]	*p* = 0.294
Imaging findings	2 [1–3]	3 [3, 4]	*p* < 0.001
Impressions/conclusions	2 [1–3]	4 [3, 4]	*p* < 0.001
Intelligibility for radiologists	2 [1–4]	4 [3, 4]	*p* < 0.001
Overall adherence with guidelines	2 [1–4]	4 [3, 4]	*p* < 0.001

Resident 1 (Table 3) assigned lower scores overall, particularly to LLM-generated reports, with a median of 2 [2, 3] for relevant clinical information and 2 [2–4] for imaging findings. Resident 2 (Table 4), while also favoring radiologist reports (*e.g.*, 5 [5] for most items), showed slightly higher tolerance toward LLM outputs, awarding them medians such as 3 [3] for clinical indications and 4 [3, 4] for impressions. Both residents found no significant difference in the description of the examination technique (*p* = 0.783 for Resident 1; *p* = 0.175 for Resident 2), suggesting this aspect was weakly addressed regardless of authorship. Overall, these findings reflect strong consensus among radiology residents regarding the superior quality and structure of human-generated reports, while also highlighting slight variability in how stringently individual readers assess LLM-generated content.

**Table 3:** Evaluation of report quality by Resident 1

Resident 1	LLMs-generated score median [range]	Radiologist-written score median [range]	Significance
Relevant clinical indications /information	2 [2, 3]	5 [4, 5]	*p* < 0.001
Examination technique and procedures	1 [1]	1 [1]	*p* = 0.783
Imaging findings	2 [2–4]	5 [4, 5]	*p* < 0.001
Impressions/conclusions	2 [2, 3]	5 [5]	*p* < 0.001
Intelligibility for radiologists	3 [2–4]	5 [5]	*p* < 0.001
Overall adherence with guidelines	3 [3, 4]	5 [5]	*p* < 0.001

**Table 4:** Evaluation of report quality by Resident 2

Resident 2	LLMs-generated score median [range]	Radiologist-written score median [range]	Significance
Relevant clinical indications/ information	3 [3]	5 [5]	*p* < 0.001
Examination technique and procedures	2 [1, 2]	2 [2]	*p* = 0.175
Imaging findings	2 [2–4]	5 [4, 5]	*p* < 0.001
Impressions/conclusions	4 [3, 4]	5 [5]	*p* < 0.001
Intelligibility for radiologists	3 [3, 4]	5 [5]	*p* < 0.001
Overall adherence with guidelines	3 [3, 4]	5 [5]	*p* < 0.001

**Fig. 1:**
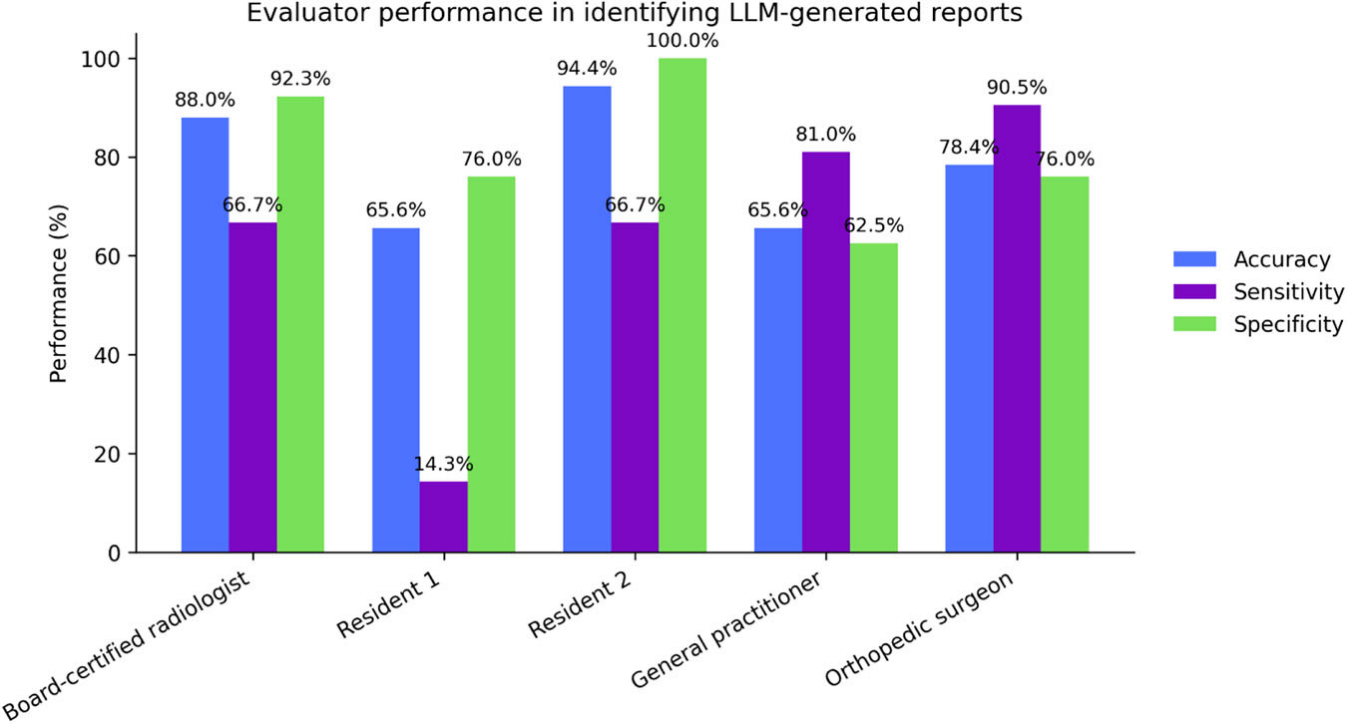
Bar chart showing the performance of each evaluator in distinguishing LLM-generated from radiologist-written lumbar spine MRI reports. Accuracy (blue), sensitivity (purple), and specificity (green) are reported for each professional profile

Among non-radiologist evaluators, both the general practitioner and the orthopedic surgeon also gave significantly higher intelligibility scores to radiologist-written reports (median 4 [4, 5] and 5 [4, 5], respectively) compared to those generated by LLMs (3 [3, 4] for both; *p* < 0.001). While there was no significant difference between the two physicians in their assessment of LLM-generated reports (*p* = 0.453), a statistically significant difference emerged for radiologist-written reports, where the orthopedic surgeon rated them higher than the general practitioner (*p* = 0.048) (Table 5). This indicates that while AI-generated reports are perceived similarly by different clinicians, human-generated reports may be more differentially appreciated depending on the clinician’s specialty (Fig. 1).

**Table 5:** Intelligibility scores assigned by the general practitioner and orthopedic surgeon for LLM-generated *versus* radiologist-written reports

Intelligibility for the physician	LLMs-generated score median [range]	Radiologist-written score median [range]	Significance
General practitioner	3 [3, 4]	4 [4, 5]	*p* < 0.001
Orthopedic surgeon	3 [3, 4]	5 [4, 5]	*p* < 0.001
Significance	*p* = 0.453	*p* = 0.048

## Discussion

This study primarily aimed to compare the correctness and completeness of lumbar spine MRI reports generated by an LLM with those written by experienced radiologists and secondarily to assess how effectively different medical professionals could identify LLM authorship. The results demonstrate that radiologist-authored reports received significantly higher ratings for the relevance of clinical information, clarity of imaging findings, diagnostic impressions and adherence to structured reporting standards. These findings confirm that human reports still provide greater contextual accuracy and domain-specific precision. However, a substantial proportion of LLM-generated reports were misclassified as radiologist-written, indicating that their style and structure can convincingly resemble human reports to non-specialist readers. Although current LLMs cannot yet match the diagnostic depth of expert radiologists, their linguistic fluency and internal consistency make them potentially useful tools for structured report drafting or clinical communication support under human supervision.

Although ChatGPT-4o did not produce overtly false or misleading statements in this study, its reports occasionally lacked the specificity and diagnostic precision expected from radiologist-authored reports. Such omissions highlight that, while the model can reproduce the structure and tone of professional reporting, it still relies on human supervision to ensure clinical accuracy and completeness.

The board-certified radiologist consistently rated radiologist-written reports significantly higher than those generated by the LLM across all major quality domains. Despite this, their ability to correctly distinguish the origin of each report was only moderate, suggesting that the stylistic and structural realism of LLM-generated content can approach expert-level reporting. This finding highlights both the progress of generative models in reproducing professional writing patterns and the ongoing vigilance of experienced readers in detecting subtle contextual inconsistencies and omissions that still betray non-human authorship.

Both radiology residents rated radiologist-written reports significantly higher across all key evaluation domains. Interestingly, no significant difference emerged in the assessment of the imaging technique description, suggesting that this section is consistently underemphasized, regardless of the report’s origin. This pattern has been noted in the literature as a common shortcoming in radiological documentation, even within structured reporting frameworks [24]. Addressing this gap could enhance both the technical transparency and the educational value of radiology reports [25].

Evaluator performance in distinguishing the origin of the reports showed marked variability across different professional profiles. Resident 2 demonstrated the highest overall accuracy, achieving perfect specificity and substantially higher sensitivity compared to other evaluators, suggesting a greater ability to recognize subtle distinctions between radiologist-written and LLM-generated reports. Conversely, Resident 1 exhibited a high false-negative rate, frequently misclassifying LLM reports as human-written. This may reflect limited exposure to structured reporting practices or a lower threshold for content acceptance. The general practitioner also struggled to differentiate between sources, often over-attributing reports to AI. Notably, the orthopedic surgeon demonstrated strong and balanced performance, characterized by high sensitivity and good specificity. This may be attributed to their frequent engagement with radiological reports in clinical decision-making. Unlike ChatGPT-generated reports, radiologist-written reports follow a format aligned with institutional norms, likely making them more interpretable for the clinician.

Among all evaluators, the orthopedic surgeon is arguably the most experienced in reading radiology reports within their own institution. Compared to radiology residents, who may adopt a more academic or technical lens, the clinician engages with each report from a practical standpoint, prioritizing its implications for patient management. As such, their feedback likely reflects the realities of day-to-day clinical practice more accurately.

While radiology reports are designed to inform both clinicians and patients, in a large specialized musculoskeletal hospital, the primary end user is often the surgeon. Reports are expected to directly support surgical planning, with less emphasis on patient-oriented communication, particularly since patients in such settings are less likely to seek care at different institutions after undergoing an MRI.

Overall clinical training, professional experience, and familiarity with radiology reporting standards influence the ability to critically evaluate the origin and quality of structured medical texts [17]. These observations align with previous studies showing that domain-specific expertise enhances the ability to detect inconsistencies and stylistic features typical of LLM-generated medical content [10]. Prior research has similarly reported that non-specialists often have difficulty distinguishing AI-generated content, underscoring the importance of contextual knowledge and reporting literacy in medical communication [8, 25].

Looking ahead, the study supports a potential role for LLMs in assisted structured reporting [26–28]. A hybrid workflow could involve the radiologist inputting key clinical data and essential imaging findings, after which the LLM generates a first draft following standardized templates and guidelines [29]. This draft would then be reviewed and finalized by the radiologist. Such a model could substantially reduce documentation time, errors [30], promote consistency in formatting and enhance clarity for referring clinicians, especially general practitioners and orthopedic surgeons. Ultimately, this approach could contribute to improved multidisciplinary communication, optimized workflow and more uniform quality in radiology reporting [31, 32].

Beyond the technical and diagnostic aspects, the progressive use of AI in radiology introduces broader educational and professional implications [33]. While LLMs can assist in report drafting and improve efficiency, their widespread adoption may contribute to a deskilling effect if radiologists increasingly rely on automated outputs without maintaining active interpretive engagement. This underscores the importance of structured reskilling programs and continuous professional development to ensure that radiologists retain critical diagnostic, communicative, and ethical competencies necessary to work safely and effectively alongside AI systems.

An evolved model could see radiologists inputting key observations into the LLMs even while the MRI scan is still underway, for instance, via voice or quick notes, enabling the system to prepare a structured draft by the time the radiologist returns to their workstation. This could streamline reporting, minimize interruptions during high-volume workflows and reduce the need to step away from the scanner room.

In line with recent reviews on the ethical deployment of LLMs in radiology, the integration of LLMs into clinical workflows must be approached with careful consideration of data governance, transparency, and model limitations. Ethical use requires that training and prompting data be fully anonymized, that outputs undergo systematic human verification, and that clinicians remain aware of the risks of hallucinations, hidden biases, and over-reliance on automated text generation [34]. As emphasized by current literature, LLMs should function strictly as assistive tools rather than autonomous decision-makers, with radiologists retaining full responsibility for diagnostic interpretation and report finalization. When embedded within a supervised workflow that includes appropriate data preparation, explicit validation steps, and clear documentation of AI involvement, LLMs can be used responsibly in healthcare while supporting efficiency and consistency without compromising patient safety or professional accountability [34].

### Study limitations

This study has several limitations that should be acknowledged. First, it was conducted in a single institution with a relatively small and unbalanced sample size, particularly in the number of AI-generated reports, which may limit the generalizability of the findings. Moreover, the number of human observers was limited, and their assessments may not fully capture the variability that could arise from a larger or different group of radiologists or physicians. Second, evaluators were aware that both radiologist-written and LLM-generated reports were included in the dataset, which may have introduced detection bias. Third, all reports were evaluated in a static and anonymized format, without integration into a real clinical workflow, potentially underestimating or overestimating their actual utility. Fourth, only one large language model (ChatGPT-4o) was assessed; results might differ with other models or future iterations. Fifth, ChatGPT-4o is not certified or formally evaluated for clinical use in medical imaging reporting. The generated reports should therefore be regarded exclusively as research outputs and not as clinical documentation. Finally, while the study included a diverse group of evaluators, it did not involve senior radiologists, whose judgments might differ from those of residents and referring clinicians.

## Conclusions

Radiologist-authored lumbar spine MRI reports demonstrated superior quality, structure, and clarity compared to those generated by an LLM. However, AI-generated reports were occasionally misidentified as human-written, particularly by non-specialist readers, suggesting that LLMs are increasingly capable of mimicking the tone and structure of professional radiology reporting. While LLMs are not yet ready to independently replace radiologists in clinical practice, these results underscore their significant potential as assistive tools.

Future integration of LLMs into radiology could follow a supervised workflow, in which radiologists input key clinical details and imaging findings and then review and refine AI-generated drafts. This hybrid approach may improve reporting efficiency, decrease administrative burden, and enhance clarity in communication with referring physicians. To support safe and effective implementation, future research should focus on optimizing prompt engineering, expanding and diversifying training datasets, and embedding AI tools within radiology reporting systems under robust clinical oversight, ensuring accuracy, reliability, and trust in their use.

## Supplementary information

ELECTRONIC SUPPLEMENTARY MATERIAL

## Data Availability

The datasets analyzed during the current study are available from the corresponding author upon reasonable request. Access to data will be provided in compliance with applicable ethical guidelines and institutional regulations.

## Abbreviations

AI: Artificial intelligence
ChatGPT: Chat Generative Pre-Trained Transformer
LLMs: Large language models
MRI: Magnetic resonance imaging
