# Supplementary material for: Can AI write reports like a radiologist? A blinded evaluation of large language model-generated lumbar spine MRI reports
Source: Eur Radiol Exp. 2026 Feb 23;10:16. doi: 10.1186/s41747-026-00682-6 (PMC12929739; doi:10.1186/s41747-026-00682-6)
Supplement: Supplementary file 1 — ELECTRONIC SUPPLEMENTARY MATERIAL [file 41747_2026_682_MOESM1_ESM.pdf]

Can AI write reports like a radiologist? A blinded evaluation of large language model–generated lumbar spine MRI reports

ELECTRONIC SUPPLEMENTARY MATERIAL

This supplementary file provides details on the prompting framework and data handling used to generate large language model (LLM)–based lumbar spine MRI reports.

Prompting procedure

All LLM-generated reports were created de novo using ChatGPT-4o (OpenAI, https://chatgpt.com/, accessed January 2025 – July 2025), based on a set of predefined clinical scenarios constructed by the research team to reflect typical indications and findings of real-world lumbar spine MRI studies. These scenarios were inspired by anonymized radiology cases and included key clinical details, imaging techniques, and representative pathological features.

All reports were generated and evaluated in Italian, which represents the standard reporting and clinical communication language in our institution. This choice was intended to reproduce realistic reporting conditions, reflecting the language, structure and workflow routinely used by radiologists in daily practice.

A consistent prompting framework was applied. Each input consisted of a brief clinical scenario and an instruction to generate a structured radiology report in Italian, organized into the following sections:

- Quesito diagnostico (Clinical indication)
- Informazioni cliniche (Clinical background and imaging technique)
- Reperti (Findings)
- Conclusioni (Conclusion)
- Raccomandazioni cliniche (Clinical recommendations)

Prompt engineering focused on clarity and consistency, using standardized language and structure across all prompts.

The evaluation was performed by five raters (one board-certified radiologist, two residents, one general practitioner and one orthopedic surgeon), all native Italian speakers with basic, non-specialized familiarity with LLMs, ensuring unbiased evaluation and comprehension consistency.

If multiple completions were returned, only the first complete and internally consistent output was retained.

Dataset compilation

The dataset included 104 consecutive lumbar spine MRI reports authored by five board-certified radiologists, each contributing approximately 20–22 cases, with 3–15 years of experience in musculoskeletal imaging. A set of 21 LLM-generated reports was created to represent a “virtual sixth radiologist” with a comparable number of cases, ensuring balanced comparisons per author/source.

Cases were selected based on report completeness, adherence to institutional formatting and clinical relevance. The sample included a representative spectrum of lumbar spine pathologies observed in routine clinical practice.

The Excel master file used for evaluation included:

- A unique identifier for each report
- The type of report (AI-generated or radiologist-written)
- Full text of the structured report

All reports were anonymized and randomized prior to evaluation to ensure blinding.

Software and tools

The following tools were used for data handling and report generation:

- Microsoft Excel for input compilation and annotation
- ChatGPT-4o (OpenAI) for generating structured report outputs

No additional browser extensions, plugins, or proprietary AI add-ons were used in the core report generation process.

Each LLM-generated report was manually reviewed by two radiologists and one researcher experienced in AI before anonymization, to verify internal consistency and to identify any hallucinations or clinically implausible statements. No hallucinations were detected in the final dataset.

The prompting framework was designed to reproduce realistic reporting conditions, meaning that the model received structured clinical information (referral reason, technique and imaging findings) analogous to what a radiologist would use when drafting a report, without access to the original images.

Before inclusion, all reports, both human-written and LLM-generated, were anonymized to remove identifying data. Quality control ensured that all reports followed the same structure and contained the required sections.

Example prompt:

"Genera un referto di risonanza magnetica della colonna lombosacrale" *(Italian version)*

"Generate a lumbar spine MRI report" *(English translation)*

Output:

Referto strutturato *(Italian version)*

Quesito diagnostico

Lombalgia cronica ingravescente. Sospetto cedimento vertebrale post-traumatico.

Informazioni cliniche

Esame eseguito mediante risonanza magnetica lombosacrale su apparecchiatura da 1.5 T. Sezioni sagittali e assiali T2-weighted, sagittali T1-weighted e STIR.

Reperti

Si osserva cedimento somatico della vertebra L1, con avvallamento della limitante somatica superiore e presenza di edema osseo nella spongiosa, suggestivo di frattura recente. Non si apprezza retropulsione significativa del muro posteriore.

I dischi intersomatici mostrano segni di disidratazione in L4-L5 e L5-S1. Presenza di protrusione discale circonferenziale in L5-S1, che impronta lo spazio peridurale anteriore senza conflitto radicolare evidente. Non si rilevano alterazioni del cono midollare.

Conclusioni

Cedimento somatico recente di L1 in esiti di trauma minore. Protrusione discale L5-S1.

Raccomandazioni cliniche

Valutazione clinica ortopedica per definizione terapeutica. Eventuale completamento con RX dinamiche in estensione/flessione per instabilità. Controllo RM a 60 giorni se persistente sintomatologia.

Structured Report *(English translation)*

Clinical Indication

Progressively worsening chronic low back pain. Suspected post-traumatic vertebral collapse.

Clinical Information

MRI of the lumbosacral spine performed with a 1.5 T scanner. Sagittal and axial T2-weighted, sagittal T1-weighted and STIR sequences were acquired.

Findings

There is evidence of vertebral body collapse at L1, with anterior superior endplate depression and bone marrow edema, suggestive of a recent fracture. No significant retropulsion of the posterior vertebral wall is observed.

Intervertebral discs show dehydration at L4-L5 and L5-S1. A circumferential disc protrusion is present at L5-S1, mildly indenting the anterior epidural space without evident nerve root compression. The conus medullaris appears normal.

Conclusion

Recent vertebral body collapse at L1 following minor trauma. Disc protrusion at L5-S1.

Clinical Recommendations

Orthopedic evaluation to define treatment strategy. Consider dynamic X-rays (flexion/extension) to assess instability. Follow-up MRI in 60 days if symptoms persist.
